# The Eurasian spruce bark beetle *Ips typographus* shapes the microbial communities of its offspring and the gallery environment

**DOI:** 10.3389/fmicb.2024.1367127

**Published:** 2024-02-16

**Authors:** Ana Patricia Baños-Quintana, Jonathan Gershenzon, Martin Kaltenpoth

**Affiliations:** ^1^Department of Insect Symbiosis, Max-Planck-Institute for Chemical Ecology, Jena, Germany; ^2^Department of Biochemistry, Max-Planck-Institute for Chemical Ecology, Jena, Germany

**Keywords:** bark beetles, microbiota, yeasts, symbiosis, transmission, *Ips typographus*

## Abstract

The Eurasian spruce bark beetle (*Ips typographus*) is currently the most economically relevant pest of Norway spruce (*Picea abies*). *Ips typographus* associates with filamentous fungi that may help it overcome the tree's chemical defenses. However, the involvement of other microbial partners in this pest's ecological success is unclear. To understand the dynamics of the bark beetle-associated microbiota, we characterized the bacterial and fungal communities of wild-collected and lab-reared beetles throughout their development by culture-dependent approaches, meta-barcoding, and quantitative PCR. Gammaproteobacteria dominated the bacterial communities, while the fungal communities were mainly composed of yeasts of the Saccharomycetales order. A stable core of microbes is shared by all life stages, and is distinct from those associated with the surrounding bark, indicating that *Ips typographus* influences the microbial communities of its environment and offspring. These findings coupled with our observations of maternal behavior, suggest that *Ips typographus* transfers part of its microbiota to eggs via deposition of an egg plug treated with maternal secretions, and by inducing an increase in abundance of a subset of taxa from the adjacent bark.

## Introduction

Bark beetles (Curculionidae: Scolytinae) are a diverse group of coleopterans that reproduce inside the tissues of their host trees. These weevils contribute to cycling of nutrients and shaping the landscape of forest ecosystems, as most of them feed on weakened, windthrown or dead trees. However, a handful of bark beetle species are capable of colonizing and killing healthy trees (Raffa et al., 2015). The rise in global temperatures has allowed these beetles to increase their number of generations per year, while a higher incidence of extreme climatic events, such as drought, have made trees more susceptible to insect attacks (Netherer et al., 2021). Moreover, the intensification of forest management in the Northern Hemisphere has resulted in higher tree densities and increased connectivity of homogeneous forest patches. These abiotic and anthropogenic factors have driven recent bark beetle population outbreaks that resulted in considerable losses of forest area worldwide (Biedermann et al., 2019). The Eurasian spruce bark beetle (*Ips typographus*) is the most aggressive pest of Norway spruce (*Picea abies*: Pinaceae). In Europe, the number of trees killed by *I. typographus* has doubled in the past decade, and the number continues to increase. In 2019 alone, the volume of timber affected by bark beetles in Europe was estimated at 70.1 Mm^3^ (Patacca et al., 2023).

Insect-microorganism associations are widespread and diverse, ranging from pathogenic to mutualistic. The insect body provides multiple habitats for microorganisms to colonize. In turn, fungi and bacteria may carry out tasks that allow the insects to thrive in challenging environments, such as providing essential nutrients, detoxifying harmful dietary molecules, producing volatiles involved in insect communication, and defending their hosts against natural enemies (Douglas, 2015). Bark beetles form symbiotic associations with fungi of diverse taxonomical groups, and, similar to ambrosia beetles, several bark beetle species have dedicated structures (i.e., mycangia) in which they harbor their fungal partners in a yeast-like phase (Francke-Grosmann, 1967). The Eurasian spruce bark beetle associates with Sordariomycetes fungi from the genera *Endoconidiophora, Ophiostoma*, and *Grosmannia*. While no mycangia are known in *I. typographus*, these beetles appear to carry fungal spores attached to pits in their elytra to vector the fungi to a new host tree (Six and Wingfield, 2011). The fungal ectosymbionts are thought to facilitate beetle attack on the tree in various ways. *Endoconidiophora polonica* may hasten tree death, since it is capable of killing healthy spruce even in the absence of bark beetles (Krokene and Solheim, 2002). Different strains of *Endoconidiophora, Ophiostoma* and *Grosmannia* may degrade tree defense metabolites (phenolic compounds) and use them as a carbon source, or metabolize them (oxygenated monoterpenes) to generate volatile cues that attract the beetles (Zhao et al., 2019; Kandasamy et al., 2023). However, the relevance of fungal ectosymbionts in promoting a successful beetle invasion has yet to be determined in natural settings (Six and Wingfield, 2011).

Although spruce bark beetle-fungi-spruce interactions have been intensively researched, the roles of other microorganisms in the invasive success of *I. typographus* remain poorly understood. Recent efforts to characterize the bacteria and fungi associated with this insect have increased our knowledge of the taxonomical composition of the fungal and bacterial communities (Fang et al., 2020; Chakraborty et al., 2023; Moussa et al., 2023; Veselská et al., 2023). However, the transmission mode of the microbiota has not been elucidated, as there is a lack of behavioral studies and quantitative data on the microbial loads present throughout the beetle's life cycle. Moreover, a large portion of experimental bark beetle research relies on laboratory-reared colonies. The effect of such rearing systems on the microbiome and the implications of possible lab-associated changes for the outcome of other experimental work on the beetles are unknown.

In this study, we used microbiological and molecular techniques to characterize the microbial communities associated with wild-collected and laboratory-reared bark beetles. We combined behavioral observations of the beetles with data on the composition and quantities of microbes to shed light on their transmission route.

## Materials and methods

### Laboratory rearing of *I. typographus*

A continuous rearing of *I. typographus* was established in the laboratory in the summer of 2021. The initial population was started with adult beetles captured in a state-owned forest near Jena, Germany (50°54'28.3"N 11°39'28.9"E). A healthy Norway spruce tree (*Picea abies*) was felled monthly from the aforementioned location and cut into 25 cm long logs. The logs' cut ends were coated in paraffin to prevent desiccation and placed individually in plastic boxes with a metallic mesh on the lid. Each log was exposed to 40 adult beetles and kept in a walk-in rearing chamber (Viessmann) with 16 h of light and 8 h of darkness at a temperature of 25°C and relative humidity of 60%. The colony has now been kept for over 14 generations.

### Isolation of bacteria from bark beetle guts and maternal oral secretions

Three larvae and three teneral adults from a wild collection, plus four mature adults from the laboratory colony were anesthetized for 1 min at −20°C, washed individually in 0.1% SDS sterile solution inside of a microcentrifuge tube, and shaken for 30 s. Then the individuals were transferred to another microcentrifuge tube with 70% ethanol and were shaken for 30 s. Subsequently, each specimen was washed thrice in sterile 1x PBS. The gut was dissected and homogenized with a sterile micropestle in 500 μL of 1 x PBS. The sample was vortexed for 10 s and then serially diluted up to 1 × 10^−4^. Each dilution was plated twice (100 μL) on lysogeny broth (LB) agar and incubated at 25°C and 28°C, respectively, for 48 to 96 h. Individual colonies were picked and streaked out three subsequent times to obtain pure cultures.

To isolate microorganisms from female oral secretions, five female beetles were collected during egg-laying from the laboratory rearing. They were gently rinsed with sterile distilled water and allowed to walk in a clean Petri dish until they were dry. A 50 μL drop of sterile 1x PBS was placed in the middle of an LB agar plate. The maternal oral secretions were collected by holding the female with sterile forceps and placing its mouthparts in contact with the PBS drop in the agar plate for 10 s−15 s. The PBS drop was plated and incubated at 28°C for 48 h to 96 h. Individual colonies were picked and streaked out three subsequent times to obtain pure cultures. These pure cultures were grown in liquid LB medium at 28°C and 300 rpm for 12 h. The resulting liquid cultures were used to create glycerol stocks that were kept at −80°C for long-term storage.

### Identification of bacterial isolates

Liquid cultures of all the isolates were prepared by dipping an autoclaved toothpick in a single colony and inoculating 7 mL of LB liquid medium in a 15 mL Falcon tube with a vented lid. The cultures were incubated for 24 h at 28°C and 250 rpm. Then, 1 ml of the culture was transferred to a 1.5 ml microcentrifuge tube and centrifuged at 16,000 g for 1 min. The supernatant was discarded and the pellet was re-suspended in 1x PBS by vortexing for 10 s. The sample was centrifuged again and the supernatant discarded. The washing step in PBS was repeated twice and the pellet was stored at −80°C until DNA was extracted. The MasterPure^TM^ complete DNA and RNA isolation Kit (Epicenter Technologies) was used following the manufacturer's protocol. The extracted DNA was used for isolate identification using the primers fD1 (5′-AGAGTTTGATCCTGGCTCAG-3′) and rP2 (5′-ACGGCTACCTTGTTACGACTT-3′) targeting the bacterial 16S rRNA gene (Weisburg et al., 1991). For amplification, 1 μl of DNA was used in a 25 μl PCR reaction with the Taq PCR Master Mix Kit (Qiagen) under the following cycling conditions: 94°C for 3 min, 35 cycles of 94°C for 60 s, 57°C for 60 s, 72°C for 60 s, and a final extension at 72°C for 10 min. The PCR products were cleaned with the DNA Clean and Concentrator-5 kit (Zymo Research) following the manufacturer's protocol and sequenced bidirectionally on a 3730XL DNA Analyser (Applied Biosystems from Thermo Fisher Scientific). Reads with minimum 700 bp and quality > 70% were used. The resulting forward and reverse sequences were trimmed by removing parts with >5% chance of error base and aligned using Geneious Prime version 2022.1 to create a consensus sequence. These consensus sequences were blasted against the NCBI database and the taxonomy was assigned to the species level when possible (>99% identity match) or to the genus level. Later, the sequences were also blasted against the 16S rRNA amplicon sequencing dataset generated in this study to confirm their presence in other sampled individuals. Isolates with no matches within the culture-independent microbiota profiling analysis were considered contaminants and removed from the isolate collection.

### Sample collection for culture-independent microbial community analysis

To characterize the microbiota of field-collected beetles, insects were collected between June and July 2021 near Jena, Germany (50°54'28.3"N 11°39'28.9"E). Mature adults and larvae were collected from five standing spruce trees. When possible, more than one gallery was sampled from each tree, ensuring that at least three individuals per life stage per gallery were present. The samples from each gallery were labeled and kept separately for future handling. In addition, 2 cm × 2 cm squares of bark were sampled from the galleries and from adjacent unattacked bark. Each sample was stored in a 15 ml Falcon tube and placed inside a cooler with ice packs to transport them to the lab.

For the microbiota profiling of laboratory-reared samples, the gallery environment (galleries, unattacked bark, pupal chambers) and beetles (mature adults, larvae, pupae, teneral adults) were sampled from generations 12 to 14 after their introduction to the laboratory colony between January and April 2022. As in the case of wild-collected samples, individuals from at least two galleries were sampled per generation and were identified accordingly for the subsequent pooling steps. A detailed overview of the sampling and replication is provided in the Supplementary Table 1. Additionally, three logs from the F14 generation were opened 5 days after the initial beetle colonization to sample the eggs and the tightly packed bark that covered the oviposition site (henceforth referred to as “the egg plug”).

The bark pieces, eggs, and egg plugs were flash frozen in liquid nitrogen and stored at −80°C until DNA extraction. The larvae and adults were anesthetized for 1 min at −20°C, washed individually in 0.1% SDS sterile solution inside of a microcentrifuge tube, and shaken for 30 s. They were then transferred to another microcentrifuge tube with 70% ethanol and again shaken for 30 s. Subsequently, each specimen was washed thrice in sterile 1x PBS and dissected as described elsewhere (Ceja-Navarro et al., 2012). The entire gut was transferred to a microcentrifuge tube, flash frozen in liquid nitrogen and stored at −80°C until DNA was extracted. The pupae underwent the same washing steps but the specimens were not dissected, as the morphology at this life stage makes it difficult to dissect the gut. Individuals carrying mites, parasitoid larvae or visible nematodes were omitted from the study.

### DNA extractions for microbial community analysis

The insect samples were homogenized in 1.5 ml microcentrifuge tubes with liquid nitrogen and sterile micro pestles. For the larvae, teneral adults and mature adults, three guts of individuals belonging to the same gallery were pooled per tube to create one biological replicate. The pupae were processed individually (a detailed overview of the sampling and replication is provided in the Supplementary Table 1). The gallery and bark samples were ground in liquid nitrogen in autoclaved mortars and pestles. A volume of ~50 μl of homogenized bark per sample was transferred to a microcentrifuge tube. For the pupal chamber samples, a volume of ~50 μl per sample was homogenized in 1.5 ml microcentrifuge tubes with liquid nitrogen and sterile micro pestles. The MasterPure^TM^ complete DNA and RNA isolation Kit (Epicenter Technologies) was used following the manufacturer's protocol, with an additional incubation step with 4 μL lysozyme (100 mg/mL) at 37°C before protein digestion. A positive control (ZymoBIOMICS™ Microbial Community Standard, Zymo Research, USA) and negative controls (extraction reagents without sample tissue) were included throughout the extraction steps. The DNA was re-suspended in Low TE buffer (1:10 dilution of TE) and the concentration was measured on a Qubit fluorometer using a 1x dsDNA High sensitivity assay (Thermo Fisher Scientific).

### Amplicon sequencing

Altogether, 55 beetle gut samples (at least 6 per life stage), 14 pupae, 3 egg samples, 3 egg plug samples, 3 pupal chamber samples, 13 bark samples, 9 gallery samples, one mock community as positive control and 4 negative extraction controls were sequenced. Bacterial 16S rRNA gene regions and fungal internal transcribed spacer 1 regions (ITS1) were sequenced by a commercial provider (StarSeq, Mainz, Germany) on a MiSeq platform (Illumina) using double indexing and a paired end approach with a read length of 300 nucleotides. The primers 341F (5′-CCTACGGGNGGCWGCAG-3′) and 806R (5′-GACTACNVGGGTWTCTAATCC-3′) were used to sequence the V3-V4 regions of the bacterial 16Ss rRNA (Klindworth et al., 2013). The primers ITS1F (5'-CTTGGTCATTTAGAGGAAGTAA-3′) and ITS2 (5′-GCTGCGTTCTTCATCGATGC-3′) (White et al., 1990; Gardes and Bruns, 1993) were used to sequence the fungal ITS1 region.

### Microbial community analysis

The reads were demultiplexed onboard in MiSeq Reporter software, allowing for one mismatch. The demultiplexed reads were processed following the DADA2 pipeline (version 1.28.0) (Callahan et al., 2016). For the bacterial sequences, the last 30 base pairs of all forward reads and the last 50 base pairs of the reverse reads were trimmed to remove nucleotides with low quality scores. For the fungal sequences, Cutadapt (version 4.0) (Martin, 2011) was used to remove the primer sequences and then a minimum sequence length of 50 base pairs was enforced to remove spurious short sequences. This different choice in the trimming approach was due to the large variability in the sequence lengths obtained in the fungal dataset (Schoch et al., 2014). For both datasets, the maximum expected error was set to 2 and the minimal overlap for merging the sequences was set to 12 nucleotides. Chimera removal was done with the “consensus” method. The resulting bacterial sequences were further filtered to remove any consensus sequence of length below 300 bp, as shorter reads were likely resulting from sequencing artifacts. The databases used for taxonomy assignment were SILVA trainset v138.1 for bacteria (Quast et al., 2013) and the UNITE general FASTA release for Fungi 2 version 8.3 (Abarenkov et al., 2021). The identities of the most abundant taxa were verified by blasting the sequence against the NCBI Nucleotide database (National Center for Biotechnology Information, 1988).

The “phyloseq” R package (version 1.44.0) (McMurdie and Holmes, 2013) was used to analyse the amplicon sequence variant (ASV) tables. Single reads and taxa that could not be classified as “bacteria” (e.g., Chloroplasts) or “fungi” were removed from the dataset. The data were not rarefied to improve the detection of differentially abundant species (McMurdie and Holmes, 2014). The alpha diversity was estimated with the Shannon and Simpson indices, and the beta diversity among groups was visualized with principal component analysis (PCoA) of the Bray-Curtis dissimilarity index. Significant differences in alpha diversity between samples were assessed with the Wilcoxon or Kruskal–Wallis rank-sum tests. Dunn's test was used to calculate pairwise comparisons between the sample types (population of origin, tissue, insect life stage) using Bonferroni's correction for multiple sample comparison. A permutational multivariate analysis of variance (PERMANOVA) was performed to evaluate the differences in beta diversity among samples. The absolute abundance data were transformed to relative counts to create the ordination plots. Core taxa shared by different life stages were identified and visualized with the R package “microbiome” (Lahti and Sudarshan, 2012). The core sequences were defined as ASVs that were present in at least 50% of the insect samples with a relative abundance of more than 0.1% per sample, respectively.

### Quantification of the bacterial and fungal titers in guts, eggs and gallery environment

The DNA obtained for the microbial community analysis was also used to estimate bacterial and fungal titers across different beetle life stages. Additional DNA extracts from eggs and egg plugs were included for this analysis: the eggs and their respective egg plugs were sampled from five galleries from two logs of the laboratory colony as described before. The eggs of each gallery were pooled together in a 1.5 ml microcentrifuge tube to create a biological replicate (~20 eggs). Their matching plugs were pooled in a separate tube. Five additional galleries were sampled to create pooled samples containing both the eggs and the plugs. DNA was extracted and measured following the same kits and protocols as the previous samples.

Quantitative PCR (qPCR) was carried out in 20 μl reactions using Blue S'Green qPCR mix (Biozym), 1 μl template DNA and 0.4 μM of each primer. The bacterial 16S primers EUB338mod (5′- TCCTACGGGAGGCAGCAG-3′) and EUB518 (5′- ATTACCGCGGCTGCTGG-3′) and fungal 18S primers FR1(5′- AICCATTCAATCGGTAIT-3′) and FF390 (5′-CGATAACGAACGAGACCT-3′) were used (Fierer et al., 2005; Chemidlin Prévost-Bouré et al., 2011).

Standard curves with defined copy numbers of the 16S rRNA gene were created by amplifying the fragment first, followed by purification and determination of the DNA concentration via NanoDrop1000 (Peqlab, Germany). After determination of the DNA concentration, eight serial 1:10 dilutions were prepared to generate the standards. One μL of each dilution was included in the qPCR reaction to standardize the measurements across reactions. The number of copies of DNA in the standard dilutions was calculated using the formula $N=\frac{DNA\;amount\;\left(ng\right)\;\times \;6.022\;\times \;10^{23}\;\left(\frac{number}{mole}\right)}{fragment\;length\;\left(bp\right)\times \;1\times 10^{9}\;\left(\frac{ng}{g}\right)\times 330\frac{g}{mole\;}}$. The DNA copy numbers in the samples were estimated taking into account the efficiency of each reaction, using the formula: $X_{0}=10^{(C_{q}-b)\cdot m^{-1}}$where *X* is the estimated DNA copy numbers, *Cq* is the quantification cycle of the qPCR, *b* is the intercept of the reaction's standard curve and *m* is the slope.

### Behavioral observations

Modified phloem sandwiches (Bedard, 1933) were used to study maternal gallery construction and egg laying behavior. In short, a 15 × 8 cm strip of phloem was peeled from a freshly cut *P. abies* log and placed between two 18 × 10 cm Plexiglas panels. The panels were sealed together with electrical insulation tape and secured with four binder clips. A male bark beetle was placed in an opening in the top panel and covered with a piece of plastic mesh. After 24 h, a female beetle was added if the male had initiated a mating chamber. Twelve successfully mated pairs were observed with an EOS 600D (Canon) camera mounted on a Stemi 2000-C microscope (Zeiss). The different life stages were photographed and egg laying behavior was recorded whenever possible.

### Statistical analysis

Significant differences in alpha diversity between samples were assessed with the Wilcoxon or Kruskal–Wallis rank-sum tests using the “biostat” package (Gegzna, 2020). Dunn's test was used to calculate pairwise comparisons between the sample types (population of origin, tissue, insect life stage) using Bonferroni's correction for multiple sample comparison. A PERMANOVA was performed to evaluate the differences in beta diversity among samples using the R packages “vegan” and “pairwiseAdonis” (Martinez Arbizu, 2017; Oksanen et al., 2022). For the microbial titers, the differences in estimated DNA copy numbers normalized by individual were assessed with a negative binomial model to correct for the data's over dispersion using the package “MASS” (Venables and Ripley, 2002). A least-squares means test (LS means) was used to assess the significant differences among sample types using the “emmeans” package (Lenth, 2023). All analyses were carried out in R version 4.3.0 (R Core Team, 2023). Figures were created with “ggplot2” (Wickham, 2009) and illustrations with BioRender.com (2023).

## Results

### Bacterial isolates from guts and maternal oral secretions

Bacterial isolates were obtained from the guts of larvae, teneral adults and mature adults, and from maternal oral secretions and cultivated on LB medium. These predominantly belonged to the class Gammaproteobacteria (Table 1). *Erwinia typographi* was the most frequently isolated bacterium, *Rahnella* species were isolated only from teneral adults, and *Pseudoxanthomonas* isolates were obtained from mature adults and maternal oral secretions. Bacilli were the second most abundant group of bacteria, with *Paenibacillus* being the dominant genus, followed by *Lactococcus*. Most of the *Paenibacillus* isolates were obtained from teneral adults. Bacteria belonging to Actinomycetia, Alphaproteobacteria, Sphigobacteriia, and Streptomycetales were also present to a lesser extent in mature adults, larvae, and female oral secretions.

**Table 1:** Number of bacterial isolates obtained per sample type and their taxonomical assignment.

	**Number of isolates per sample type**				
**Taxonomic assignment**	**Mature adults**	**Teneral adults**	**Larvae**	**Female oral secretions**	**Total**
**Actinomycetia**	**4**			**3**	**7**
*Microbacterium azadirachtae*	1				1
*Microbacterium flavum*	1			1	2
*Microbacterium* sp.	1			1	2
*Micrococcus yunnanensis*	1				1
*Streptomyces anthocyanicus*				1	1
**Bacilli**	**3**	**22**	**3**		**28**
*Bacillus cereus*			1		1
*Brevibacillus agri*			1		1
*Lactococcus cremoris*	2				2
*Lactococcus lactis*		3			3
*Lactococcus* sp.		1			1
*Paenibacillus* sp.	1	13			14
*Paenibacillus typhae*			1		1
*Paenibacillus xylanilyticus*		5			5
**Sphingobacteriia**	**3**		**1**		**4**
*Sphingobacterium* sp.			1		1
*Sphingobacterium detergens*	2				2
*Sphingobacterium multivorum*	1				1
γ**-Proteobacteria**	**14**	**10**	**15**	**11**	**50**
*Erwinia* sp.			4		4
*Erwinia* sp.			3		3
*Erwinia typographi*	10	3	8	5	26
*Pseudoxanthomonas sp*.				4	4
*Pseudoxanthomonas spadix*	3			1	4
*Rahnella aquatilis*		1			1
*Rahnella* sp.		2			2
*Rahnella variigena*		3			3
*Rahnella woolbedingensis*		1			1
*Rouxiella silvae*				1	1
*Stenotrophomonas maltophilia*	1				1
α**-Proteobacteria**	**1**				**1**
*Ochrobactrum* sp.	1				1

### Bacterial community composition and structure

**Figure 1:**
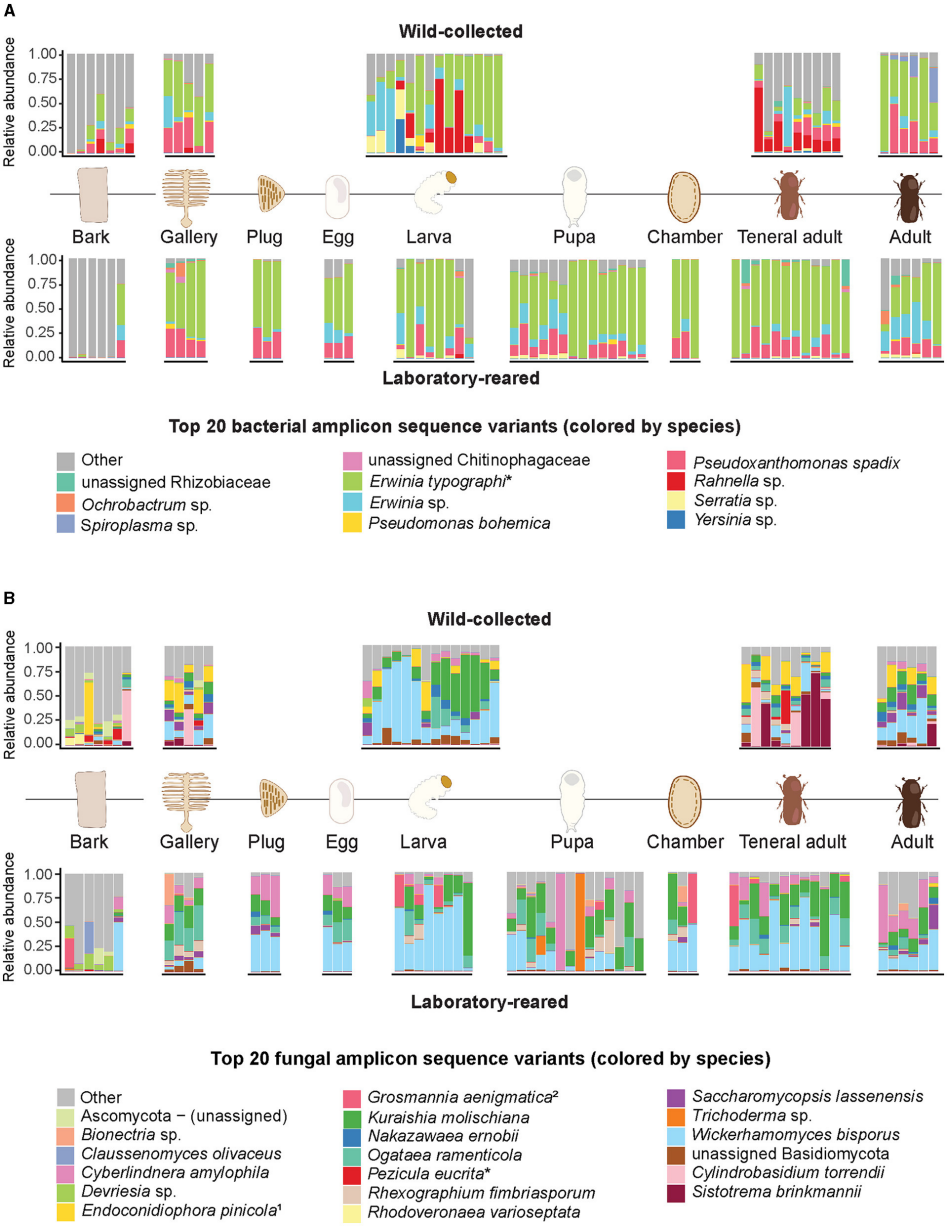
Relative abundances of the 20 most abundant bacterial and fungal amplicon sequence variants across wild-caught and laboratory-reared life stages of *I. typographus* and their environment. **(A)** Bacteria, **(B)** fungi. Relative abundances are indicated at species level; taxa marked with an asterisk (^*^) were refined to species by nucleotide blast against the NCBI database. When blasted against NCBI database, the assigned taxonomies were ^1^*Endoconidiophora polonica* and the anamorph ^2^*Leptiographium piceaperdum*.

**Figure 2:**
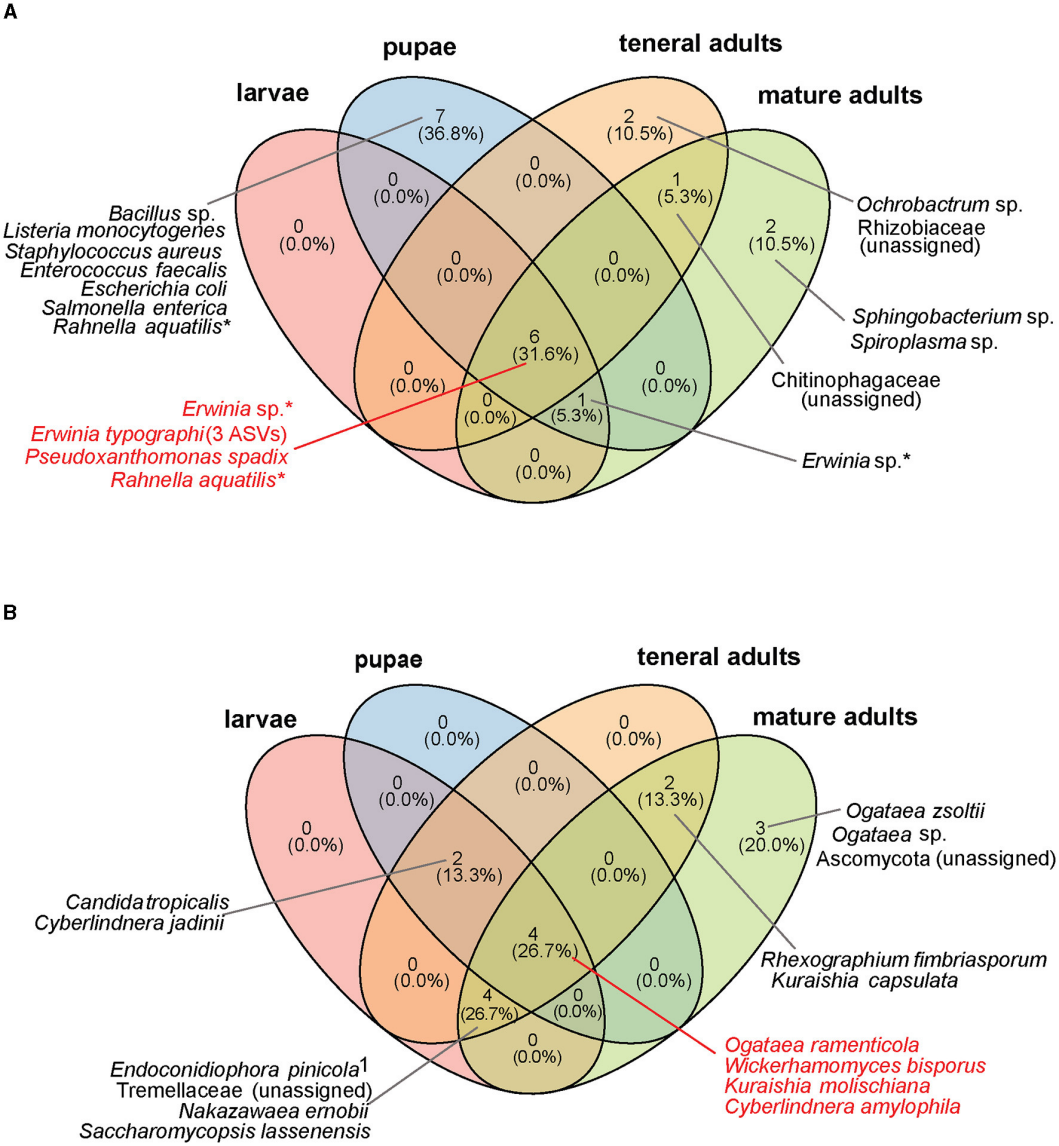
Core microbial taxa shared by different life stages of *I. typographus* in both laboratory-reared and wild-collected insects. **(A)** Core bacterial community, **(B)** core fungal community. The core taxa were defined as ASVs present in ≥ 50% of all samples, with a relative abundance ≥ 0.1% per individual. Taxa marked with an asterisk (*) are present in multiple life stages at the species level, but a particular ASV was identified in each life stage. ^1^Further identified as *Endoconidiophora polonica* when blasted against the NCBI database.

In the culture-independent high-throughput profiling, Gammaproteobacteria largely dominated the bacterial communities associated with the beetle gut across all the life stages, as well as the eggs and the beetle-modified environment. The most abundant taxon was *E. typographi*, followed by *Pseudoxanthomonas spadix* and *Rahnella* sp. All three taxa were present in both wild-collected and laboratory-reared specimens, but the wild-collected samples had a higher prevalence of *Rahnella* sp., while *P. spadix* was more common in the laboratory population. Additionally, a further *Erwinia* ASV (not identifiable to the species level) was abundant in most of the beetle and the beetle-modified environment samples. Even though these Gammaproteobacteria were also present in the unattacked bark samples, they were not as abundant as in the beetle guts, eggs, and gallery environment (Figure 1A). *E. typographi, P. spadix, Rahnella* sp. and *Erwinia* sp. formed the core gut bacterial community shared by all life stages across the wild-collected and laboratory-reared samples (Figure 2A). *Serratia* sp., *Pseudomonas bohemica, Ochrobactrum* sp., and an unidentified ASV belonging to Rhizobiaceae were present to a lesser extent in the galleries, larvae, pupae, teneral adults and mature adults of both populations. An unidentified Chitinophagaceae ASV was present in the galleries and some of the adults. The mollicute *Spiroplasma* sp. was identified in variable abundances in all the mature adults captured in the wild, but it was almost absent from the laboratory-reared individuals. Overall, the composition and structure of the bacterial communities associated with *I. typographus* and the gallery environment were largely different from the ones in the unattacked bark. The unattacked bark harbored taxa belonging mostly to Alphaproteobacteria, Gammaproteobacteria and Verrucomicrobiota (Supplementary Figure 2A).

### Fungal community composition and structure

Saccharomycetales yeasts largely dominated the fungal communities across all beetle life stages, as well as the gallery environment (Figure 1B). *Wickerhamomyces bisporus* was ubiquitous, followed in abundance by *Kuraishia molischiana, Ogataea ramenticola* and *Cyberlindnera amylophila*. These four yeasts formed the core gut community of all life stages across the wild-collected and laboratory-reared samples (Figure 2B). *Nakazawaea ernobii* and *Saccharomycopsis lassenensis* were also widespread among different life stages but their relative abundances were lower when compared to the core yeasts. The second most abundant order of fungi were the Sordariomycetes. Interestingly, the abundance of *Endoconidiophora polonica*, one of the best known fungal ectosymbionts of *I. typographus*, was higher in the samples from the wild population than in the laboratory-reared beetles and galleries. Another ASV, assigned to *Grosmannia aenigmatica*, was abundant in some of the larvae, pupae, pupal chambers and teneral adults of the laboratory-reared samples. A search of this ASV against the NCBI database revealed multiple 100% sequence identity hits with *Leptiographium piceaperdum* (the anamorph of *G. piceaperda*). A third sordariomycete fungus, *Rhexographium fimbriasporum*, was present mostly in the laboratory-reared insects. Additionally, *Sistotrema brinkmannii* and *Cylindrobasidium torrendi* (Basidiomycetes) were very abundant in the wild-caught teneral adults. An unidentified Basidiomycete appeared in several gallery and insect samples from the wild and, in lower abundances, in the laboratory-reared samples. As observed in the bacterial communities, the composition and structure of the fungal communities associated with the unattacked bark were remarkably distinct from the ones present in the beetle guts, eggs, and gallery environment. The unattacked phloem harbored taxa belonging mostly to Sordariomycetes, Dothideomycetes, Leotiomycetes and Lecanoromycetes (Supplementary Figure 2B).

### Bacterial and fungal diversity

The 16S rRNA dataset had an average of 14107 reads with a minimum of 6 and a maximum of 48820 across samples. The ITS dataset had an average of 48030 reads with a minimum of 12 and a maximum of 334941 across samples. The bacterial alpha diversity of the unattacked bark was significantly higher than that of all other samples (Kruskal–Wallis test: Shannon χ^2^ = 40.03, *p* < 0.0001; Simpson χ^2^ = 38.196, *p* < 0.0001; Supplementary Figure 1B) and did not differ significantly between wild-collected and laboratory-reared samples (Wilcoxon test: Shannon *p* = 0.093, Simpson *p* = 0.079; Supplementary Figure 1A). Similar to the case of bacteria, fungal alpha diversity was also higher in the bark and in the galleries than in the beetle guts, eggs, pupal chambers, and bark plugs placed over the eggs immediately after oviposition (Kruskal–Wallis test: Shannon χ^2^ = 48.673, *p* < 0.0001; Simpson χ^2^ = 37.234, *p* < 0.0001; Supplementary Figure 1D). In contrast, the wild-collected samples showed a significantly higher fungal alpha diversity than the ones from the laboratory colony (Wilcoxon test: Shannon index *p* = 0.002, Simpson index *p* = 0.02, Supplementary Figure 1C).

**Figure 3:**
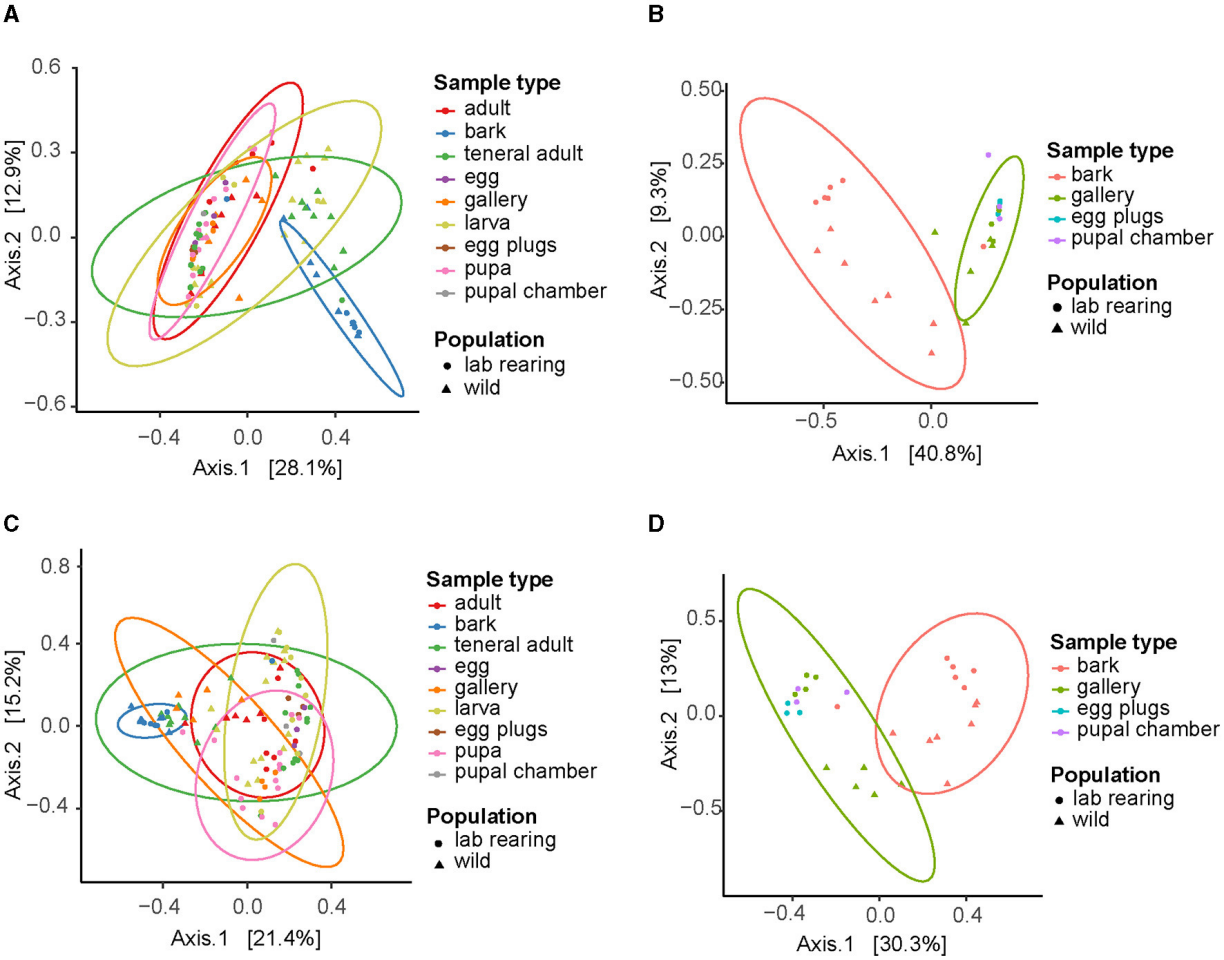
Beta diversity of bacterial and fungal communities associated with *I. typographus*. Principal Component Analysis (PCoA) plots of the Bray-Curtis distances for **(A)** bacterial taxa in all sample types, **(B)** bacterial taxa in bark and beetle-modified environment, **(C)** fungal taxa in all sample types, **(D)** fungal taxa in bark and beetle-modified environment.

The beta diversity analysis revealed significant differences in the microbial community composition across beetle life stages. The principal component analysis (PCoA) of both the bacterial and fungal Bray-Curtis distances showed the unattacked bark clustering separately from the rest of the samples (Figures 3A, C), and the PERMANOVA generated significant results for the bacterial (*F* = 3.215, *p* = 0.001) and fungal (*F* = 4.227, *p* = 0.001) Bray-Curtis metrics. Further inspection of the data revealed a clear separation of the bark samples from the beetle-modified environment (galleries, egg plugs, and pupal chambers) (Figures 3B, D, Supplementary Tables 2, 3). The beta diversity analysis across life stages did not reveal significant differences in community composition except for the pupae, both in the bacterial (*F* = 2.636, *p* = 0.001) and the fungal communities (*F* = 2.902, *p* = 0.001) (Supplementary Figures 5A, B, Supplementary Tables 4, 5). PERMANOVA analysis showed significant differences between the bacterial (*F* = 6.1837, *p* = 0.001) and fungal (*F* = 12.100, *p* = 0.001) communities of the samples from the wild collection and the laboratory rearing, but no clear patterns were observed in the PCoA (Supplementary Figures 5C, D).

### Bacterial and fungal titers across life stages

**Figure 4:**
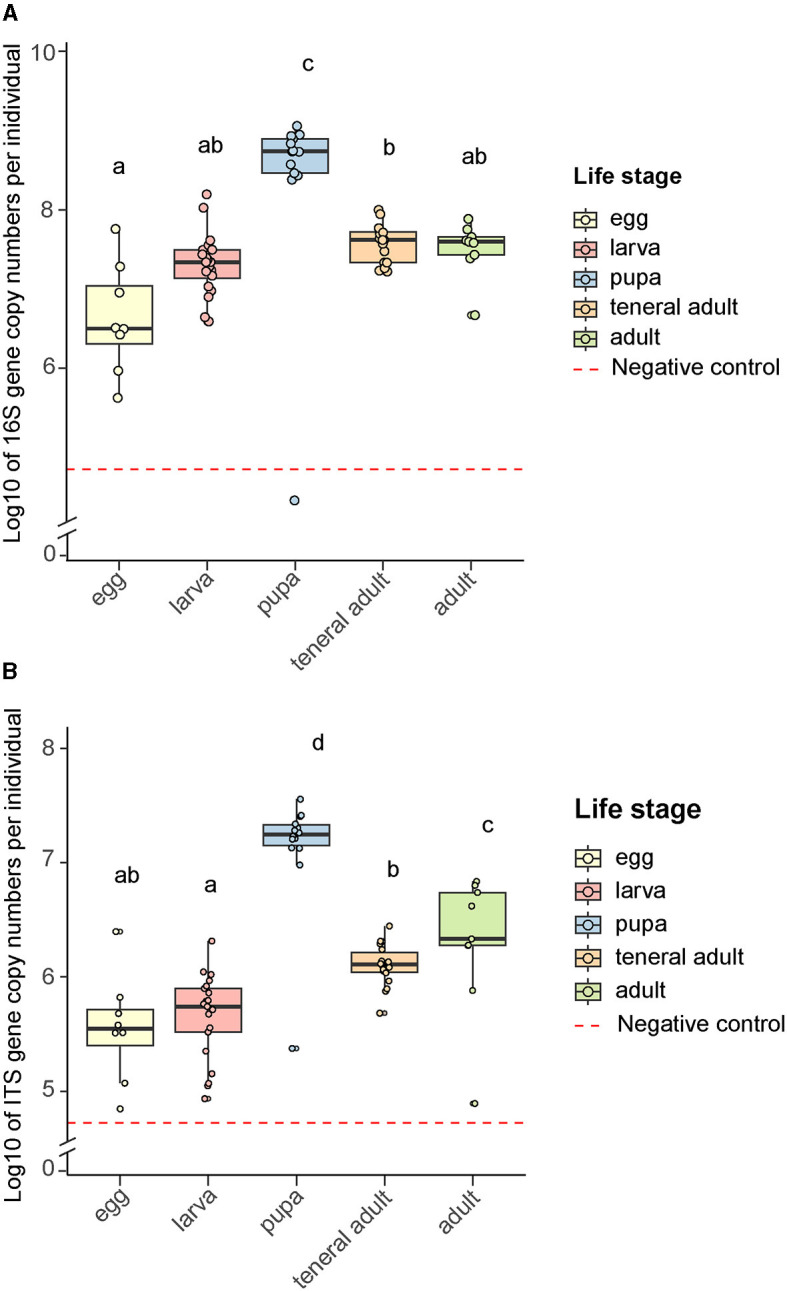
Microbial rRNA gene copy numbers across bark beetle life stages. **(A)** Bacterial 16S gene copy numbers, **(B)** Fungal ITS gene copy numbers. Letters indicate significant differences among groups (least-squares means pairwise comparison with Tukey adjustment, *p* < 0.05). Data were log-transformed to facilitate their visualization. Negative controls were samples without any insect or plant tissue, but extraction reagents only, that were processed and subjected to quantitative PCR in the same manner as other samples.

The bacterial loads in the gut did not differ significantly across life stages except for the pupae, where the entire individual including the gut was sampled. The copy number of the 16S gene was higher for the pupae than the mean copy number for the first instar larvae, teneral and mature adults (Negative Binomial GLM *p* = 1.98 × 10^−11^, LS means *p* < 0.0001, Figure 4A). Due to the pooling of egg samples, it is difficult to assess the microbial load on individual eggs accurately. Assuming an average of 20 eggs per sampled gallery, the bacterial load of an individual egg was lower than that in the gut of the other life stages, which was not statistically significant except for the teneral adults (Negative Binomial GLM *p* = 0.00718, LS means *p* < 0.0081). When compared to their matching plugs, the eggs themselves had higher numbers of 16S rRNA gene copies (Negative Binomial GLM *p* = 0.0046, LS means *p* < 0.0127, Supplementary Figure 4A). The bacterial titers in the additional samples where the eggs and plugs were collected together were not statistically different from the egg-only samples, suggesting that the contribution of the plugs to the bacteria in the oviposition site is lower than the contribution from the bacteria present on the egg surface (Negative Binomial GLM *p* = 0.0.094, LS means *p* < 0.215). The 16S rRNA gene copy numbers were an order of magnitude higher in the galleries than in the unattacked phloem (Negative Binomial GLM *p* = 0.00104, LS means *p* < 0.003, Supplementary Figure 3A). The same trend was observed for the pupal chambers, but was not statistically significant (Negative Binomial GLM *p* = 0.05507, LS means *p* < 0.1335).

The fungal load in the gut remained stable as the life stages progressed (Figure 4B). As in the case of bacteria, the pupae were the exception, as they had higher titers than the rest of the life stages (Negative Binomial GLM *p* = 5.1 × 10^−8^, LS means *p* < 0.0001). As opposed to bacteria, the egg plugs carried significantly higher ITS gene copy numbers than the eggs (Negative Binomial GLM *p* = 2.99 × 10^−9^, LS means *p* < 0.0001, Supplementary Figure 4B). The fungal titers in the samples where eggs and plugs were collected together were also higher than in the egg-only samples (Negative Binomial GLM *p* = 2.75 × 10^−10^, LS means *p* < 0.0001), which indicates that the plugs make a major contribution to the presence of fungi in the oviposition site. As for the bacteria, the fungal load was an order of magnitude higher in the galleries than in the unattacked phloem (Negative Binomial GLM *p* = 0.00289, LS means *p* < 0.0081, Supplementary Figure 3B). This was also the case for the pupal chambers, which had a significantly higher number of ITS gene copies than the unattacked phloem (Negative Binomial GLM *p* = 0.00594, LS means *p* < 0.0164) and did not differ in fungal titers from the galleries (Negative Binomial GLM *p* = 0.00594, LS means *p* < 0.9260).

### Observations on female oviposition behavior

**Figure 5:**
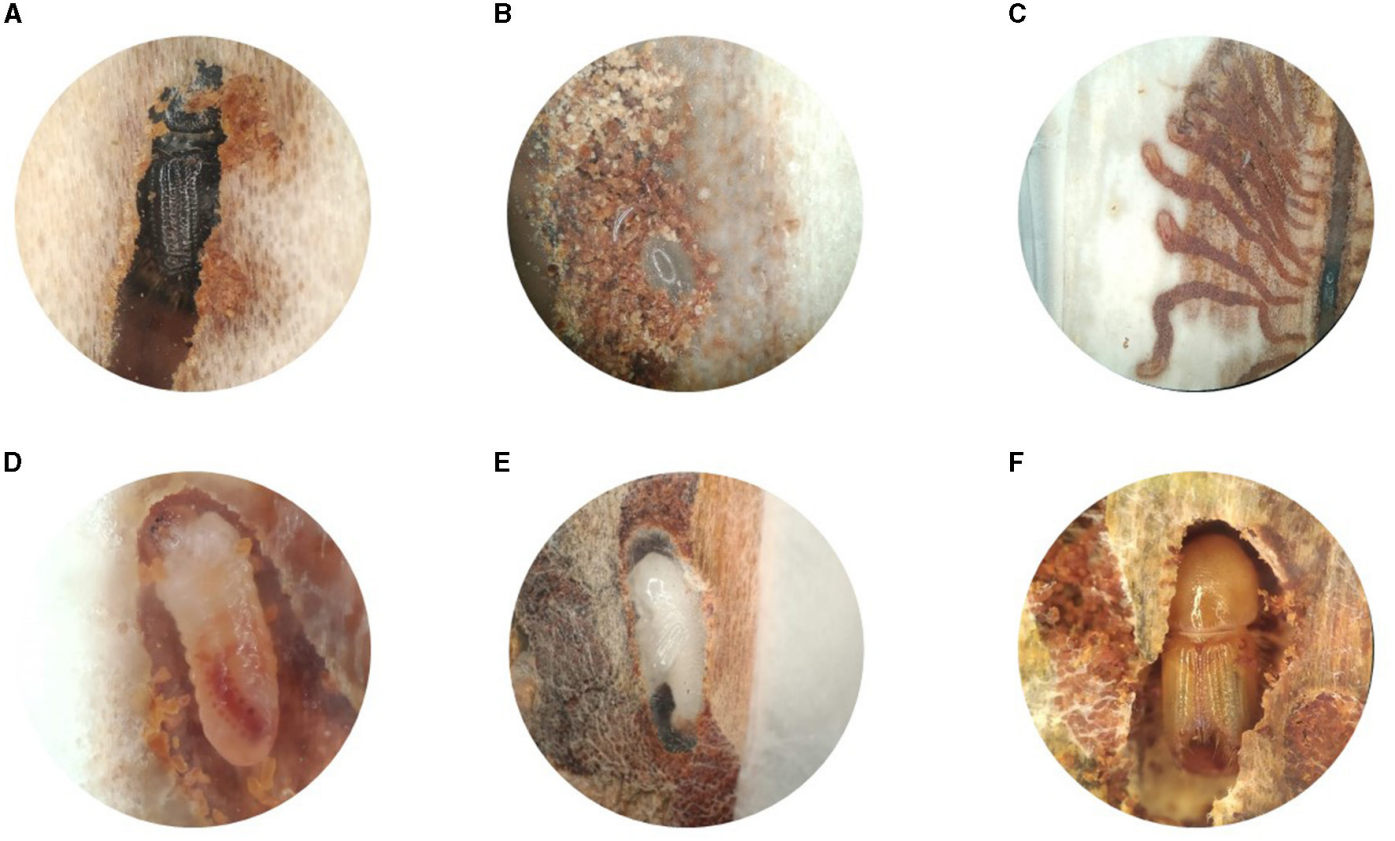
Development of *Ips typographus* inside the phloem of *Picea abies*. **(A)** Adult female in the maternal gallery. **(B)** Detail of an egg in its niche, protected by a plug of chewed bark. **(C)** Individual larval galleries radiating from the oviposition site. **(D)** Close-up of a second instar larva. **(E)** Pupa inside the pupal chamber lined with frass. **(F)** Teneral adult during maturation feeding.

To explore the likelihood of vertical transmission of microbes from parents to offspring, we first used phloem sandwiches to observe parental behavior, offspring development, and feeding habits of the different life stages (Figure 5). The gallery excavation and developmental stages have been extensively documented in previous works [as summarized by Schebeck et al. (2023)]. Earlier literature indicates that the parent beetles “groom” the eggs, but a detailed description of this behavior is missing. We observed that, while boring the maternal gallery, the female removed most of the bark debris by pushing it toward the mating chamber with its elytral declivity. However, it stored part of the chewed bark at the farthest end of the tunnel. Every 2–3 millimeters, the female stopped boring the main gallery and carved a niche with its mouthparts on one of the sides. Once the niche was large enough to harbor an egg, the mother traveled back to the mating chamber to make a 180° turn. With the abdomen facing the end of the maternal gallery, it laid an egg in the niche. It then rapidly moved back to the mating chamber to turn around, and returned to the freshly laid egg at a considerable speed. Using the chewed bark stored at the end of the gallery, the mother quickly created a bark plug to cover the egg (Supplementary Video 1, Figures 5A, B).

## Discussion

In this study, we characterized the microbial communities associated with the gut and galleries of the European spruce bark beetle (*I. typographus*) across its life stages. Additionally, we carried out behavioral observations to record possible modes of symbiont acquisition and transmission, as well as quantitative PCR to assess the changes in microbial loads throughout the beetle's life cycle.

Microbial amplicon sequencing analyses showed that the composition and structure of the bacterial communities were stable throughout the beetle's development and broadly overlapping between wild-caught and laboratory-reared beetles (Figures 1A, B). However, some of the bacterial taxa found in the field-collected beetles were lost or diminished in most of the individuals raised in the laboratory, such as *Spiroplasma*, certain *Rahnella* ASVs, and *Yersinia* (Figure 1A). Similarly, the fungal communities associated with field-collected vs. laboratory-reared beetles showed considerable overlap, but also distinct differences. The ectosymbiont *E. polonica*, while present in most wild-collected samples, was generally absent from the laboratory-reared ones. In turn, an ASV assigned to the fungus *Leptiographium piceaperdum* (the anamorph of *G. piceaperda*) was present in high abundance in some of the laboratory individuals and largely absent from the wild population. While it may be possible that this fungus is present in the beetle population and the better known ectosymbiont *G. penicillata* is absent, an alternative explanation may lie in the limitations of the primers we used for amplicon sequencing. Combining the ITS data with sequences from the β-tubulin and the ribosomal large subunit (LSU) genes may offer a better resolution at species level in Ophiostomatoid fungi (Zipfel et al., 2006). A subset of saprophytic Basidiomycetes (*C. torrendii* and *S. brinkmanii*) were abundant in the wild teneral and mature adults, but largely absent from the laboratory-reared beetles. Reduction in diversity and loss or replacement of some gut symbionts can be expected when bark beetles are kept in captivity (Dohet et al., 2016). Stable temperature and humidity, a reduced number of interactions with other invertebrate species, and the sanitization practices in our rearing could explain these differences. A comparable phenomenon is observed in industrial pollinator rearing, where minor gut symbionts are lost or replaced in artificially-reared bumblebee colonies, but the core taxa remain (Meeus et al., 2015).

Wild-collected and laboratory-reared insects shared several of the most abundant gut bacteria (*E. typographi, P. spadix, Rhanella aquatilis*, and *Erwinia* sp.; Figure 2A) and fungi (*W. bisporus, O. ramenticola, K. molischiana*, and *C. amylophila;* Figure 2B) throughout their development. These taxa have previously been reported in *I. typographus* at different life stages (Chakraborty et al., 2023; Veselská et al., 2023), across geographic locations (Chakraborty et al., 2023; Moussa et al., 2023) and seasons (Moussa et al., 2023; Veselská et al., 2023), and regardless of whether the beetle populations were experiencing an endemic or an epidemic phase (Moussa et al., 2023). The consistency of these associations throughout time and space indicates the existence of a common core community in the *I. typographus* gut (Risely, 2020).

This core microbiome was also dominant in the gallery environment. In fact, our beta-diversity analysis showed that the communities in the beetle tissues clustered together with those of the galleries, egg plugs and pupal chambers but were significantly different from those in the bark (Figure 3). In addition, the overall bacterial alpha-diversity was higher in the bark than in the gallery environment or the beetle guts (Supplementary Figure 1B), and the bark communities were characterized by high abundances of Alphaproteobacteria, Verrucomicrobiota, Acidobacteria and, to a lesser extent, Gammaproteobacteria (Supplementary Figure 2A). Indeed, the core taxa *E. typographi, P. spadix, Rhanella aquatilis*, and *Erwinia* sp. were present in the bark, but at much lower relative abundances than in the beetle samples or galleries (Figure 1A). The fungal alpha-diversity was also higher in the bark than in the beetle guts (Supplementary Figure 1D), and the communities were dominated by Sordariomycetes, Dothideomycetes, Leotiomycetes and Lecanoromycetes (Supplementary Figure 2B). As noted earlier, the beetle core yeasts and the fungal ectosymbionts were seldom found in the unattacked phloem. The occasional identification of these fungi in the intact phloem may be due to inconsistencies in sampling or contamination and not necessarily to the fungal taxa being already present before the arrival of the colonizing adults. The present study and two earlier surveys (Chakraborty et al., 2023; Veselská et al., 2023) used the bark adjacent to the galleries as a control and omitted unattacked trees, which could have given greater certainty about the composition of the microbial communities in the absence of a beetle attack. The similarities among the communities in beetle samples and their gallery environment, along with the distinct bacterial and fungal profile of the bark, indicate that the beetle modifies the microbial community of the substrate it inhabits and feeds on.

By raising beetles in the laboratory in “phloem sandwiches,” strips of phloem tissue sealed in a Plexiglas chamber, we observed that female *I. typographus* provided each egg with a protective plug made from masticated phloem (Figures 5A, B, Supplementary Video 1). This has been described previously as “egg grooming” and is part of a series of subsocial behaviors shared by *I. typographus* and other related bark beetle species (Schebeck et al., 2023). The 16S amplicon sequencing data showed that *E. typographi, P. spadix*, and an unassigned *Erwinia* species dominated the bacterial communities of the eggs and the plugs (Figure 1A). This was consistent with the culture-dependent bacterial characterization, where most of the isolates obtained from the maternal oral secretions belonged to *E. typographi* and *Pseudoxanthomonas* spp. as well as *Microbacterium* spp. (Table 1). The ITS amplicon sequencing data revealed that the dominant fungi on the eggs and plugs were the yeasts *W. bisporus, O. ramenticola, K. molischiana*, and *C. amylophila* (Figure 1B). The communities of the eggs and plugs differed significantly from those of the unattacked bark (Figures 1A, B) and matched the core bacterial and fungal taxa shared by all life stages (Figures 2A, B). Additionally, microbial loads on the eggs and plugs were similar or higher than those in the larval guts (Figure 4, Supplementary Figure 3). These results indicate that females promote the presence of the core gut symbionts on the egg and the oviposition site. Further, the quantitative data support the hypothesis of maternal transmission of fungi via the egg plugs, which carry the highest fungal load in the oviposition site (Supplementary Figure 4A).

Vertical extracellular transmission is well documented across different insect orders, where females pass on the symbionts to the next generation by inoculating their eggs or the oviposition sites (Salem et al., 2015). In Coleoptera, the roles of these microbial partners range from chemically defending the eggs to supplementing the diet of the newly hatched larvae with essential nutrients. For example, females of the tenebrionid *Lagria villosa* house *Burkholderia gladioli* bacteria in two accessory glands connected to their reproductive system. The female smears these bacteria onto the egg surface during oviposition, where the symbionts produce antimicrobial compounds that defend the insect's eggs against fungal pathogens (Flórez et al., 2017). Some beetles transmit their symbiotic partners to their offspring in specialized packaging. This is the case for the tortoise beetle *Cassida rubiginosa*, which deposits its obligate *Stammera* symbiont packed in a caplet on top of each egg. This extracellular symbiont improves the beetle's diet by supplying its host with pectinolytic enzymes (Salem et al., 2017). Other coleopterans rely on the transmission of yeast symbionts to ensure the survival of their offspring in nutrient-poor diets, such as the longicorn beetle *Leptura ochraceofasciata* (Kishigami et al., 2023), and the lizard beetle *Doubledaya bucculenta* (Toki et al., 2012). In all of the above, the female harbors a single bacterial or fungal taxon in a specialized organ and inoculates the egg during oviposition.

Our results show that *I. typographus* females promote a group of taxa in the oviposition site rather than transmitting a single symbiont. While no dedicated symbiotic organs (i.e., bacteriomes, mycangia or specialized glands) have been reported in *I. typographus* to date, the maternal oral secretions may serve as a source of additional bacterial inoculum for the plugs and eggs. Similar behaviors have been recorded in the North American spruce beetle *Dendroctonus rufipennis*: when challenged by fungal entomopathogens, the adults spread oral secretions around their bodies and galleries (Cardoza et al., 2006). These secretions contain Proteobacteria, Actinobacteria, Bacteroidetes, and Firmicutes that are able to inhibit the growth of the noxious fungi to different degrees. Subsequent work revealed that the mountain pine beetle *Dendroctonus ponderosae* and the pine engraver *Ips pini* also harbor closely related bacteria in their oral secretions (Cardoza et al., 2009). Among the isolates we obtained from the mouthparts of female *I. typographus*, we identified two *Microbacterium* and one *Streptomyces* (Table 1), genera that were able to inhibit the growth of *Aspergillus fumigatus* and *Trichoderma harzianum* in the North American spruce beetle (Cardoza et al., 2006). Even if these taxa were not among the dominant members of the bacterial communities of the eggs and plugs, they may be able to produce antifungal compounds that contribute to protecting the offspring from opportunistic entomopathogens. Of the dominant taxa present in the oviposition site, multiple *E. typographi* strains isolated from *I. typographus* have been shown to fully or partially inhibit the growth of *Beauveria* spp., *Metarhizium anisopliae, Lecanicillium muscarium* and *Isaria* spp. in *in vitro* assays (Peral-Aranega et al., 2023). Thus, bacteria associated with *I. typographus* may have the potential to protect the beetles and their galleries from detrimental fungi.

Aside from dedicated organs, fungi can attach to less specialized structures on the insect's exoskeleton, such as elytral pits (Furniss et al., 1990; Bleiker et al., 2009; Kandasamy et al., 2023) or setae (Brysch-Herzberg, 2004). Some of the Saccharomycetes detected were present in the unattacked phloem samples, but their abundance was significantly lower than in the eggs, beetle guts, and gallery environment (Figure 1B). This is consistent with previous studies, where the authors found low (Veselská et al., 2023) or near-zero (Chakraborty et al., 2023) relative abundances of yeasts in the phloem adjacent to the galleries. This shift in abundance is another indicator of microbial community manipulation by the female beetles in the oviposition site.

Veselská et al. (2023) suggest that the beetles acquire their gut communities strictly horizontally from the diet. Certainly, the core gut and gallery bacteria are already present in the unattacked phloem and the beetles might acquire them through feeding. However, some selected bacterial taxa, along with beetle-associated yeasts, are enriched in the eggs and the plugs by the females, which makes the inoculum available on the egg surface (Figure 1A). The parents likely vector the yeasts and filamentous ectosymbionts into the phloem by carrying them attached to their setae and elytral pits (Kandasamy et al., 2023). The feces may also serve as a source of inoculum from the beetle gut to the gallery environment. Subsocial and eusocial insects are considerably more exposed to their own frass than solitary insects, and in some cases, the fecal microbiome is crucial in protecting their nest or gallery environment from pathogens (Cole et al., 2021; Pessotti et al., 2021). Therefore, we hypothesize that rather than a strict horizontal transmission, the females inoculate the offspring's environment with their core microbial associates and set favorable conditions for the proliferation of potentially beneficial symbionts from the bark. The burying beetle *Nicrophorus vespilloides* displays a similar behavior, using oral and anal secretions to transmit their gut microbiota to the carcasses they colonize. The parent beetles regulate the bacterial and fungal communities on the carcass to preserve this ephemeral food resource for their offspring during the larval stages (Vogel et al., 2017). We propose a mixed mode of transmission for *I. typographus* as well, with vertical transmission of microbes via the egg surface and egg plugs as well as the gallery environment, in addition to horizontal acquisition from the bark. This has not only been suggested for other bark beetles (Rivera et al., 2009), but is also the predominant mode of microbiota transmission in vertebrates and invertebrates (Ebert, 2013).

Subsocial insects structure their microbial communities through parental care, selective feeding, collective feeding, direct management of the microbes (e.g., applying secretions), and vertical transmission (Biedermann and Rohlfs, 2017). The European spruce bark beetle displays all of these strategies (i.e., grooming the eggs, feeding on a narrow host range, mass-attacking their host tree with the aid of aggregation pheromones, protecting the egg niche with a plug, and applying oral secretions loaded with bacterial and fungal cells on the oviposition sites), resulting in a stable core community. The association with Gammaproteobacteria in the gut and galleries is common in other bark and ambrosia beetles, as well as in other conifer-feeding coleopterans in which no fungal partners are known (Delalibera et al., 2007; Morales-Jimenez et al., 2009, 2012; Berasategui et al., 2016; Dohet et al., 2016; Briones-Roblero et al., 2017; Hernandez-Garcia et al., 2018; Barcoto et al., 2020; Chakraborty et al., 2020). This widespread pattern points toward the importance of dietary acquisition for shaping the microbiota in these insects.

In addition to transgenerational transmission, holometabolism poses several challenges for the persistence of insect microbiota. The gut undergoes considerable changes during complete metamorphosis. The larvae eliminate most of their gut contents before pupation, and the rest is packaged in the form of the meconium that remains in the peritrophic matrix of the pupae and is expelled from the adult body during ecdysis. This eliminates a large portion of the gut bacteria, but microorganisms can persist in bacteriocytes, specialized crypts or pouches (Nardi et al., 2006; Engel and Moran, 2013; Hammer and Moran, 2019). However, contamination of the adult gut with the larval gut microbiota can also occur without such structures. In some cases, an interplay between the insect's immune response and the microbe's competitive abilities ensures the persistence of the gut microbiota from larvae to adults (Johnston and Rolff, 2015). In *I. typographus*, the bacterial and fungal titers increased during pupation (Figure 4). These patterns may be explained by the larvae accumulating microbes during development by feeding, resulting in high titers in the third-instar larvae (not sampled) and pupae. The pupae may then shed most of the microorganisms, resulting in lower titers in the adults. Since we did not dissect the pupal guts, it is also possible that other parts of the body serve as a reservoir of bacteria and fungi, e.g., the presence of yeasts in pupal integuments has been recorded in several bark beetle species (Davis, 2015). Localizing the microorganisms in the pupae with microscopy would clarify whether this is the case.

The stability of the gut microbiota could be an indicator of its functional relevance to the host (Barcoto et al., 2020). Genomic and *in-vitro* approaches have revealed multiple possible contributions of fungal associates to the success of *I. typographus* in colonizing well-defended host trees. For example, bark beetle-associated yeasts have metabolic capabilities that could directly benefit the insects, such as the production of anti-aggregation pheromones (Leufvén et al., 1984; Hunt and Borden, 1990), the emission of attractants (Brand et al., 1977), the assimilation of carbohydrates (Rivera et al., 2009), and the breakdown of cellulose, chitin and lipids (Cheng et al., 2023). Further, the genomes of the yeasts *K. molischiana, Cryptococcus* sp., *Nakazawaea ambrosiae, O. ramenticola*, and *W. bisporus* contain complete pathways for essential amino acid and vitamin B6 biosynthesis (Cheng et al., 2023), and so could be directly involved in improving the quality of the insect diet (Stefanini, 2018). Similarly, the filamentous fungi have been suggested to play important roles in bark beetle ecology by exhausting the tree defenses and providing the beetle with nutritional benefits (Six and Wingfield, 2011). Ophiostomatoid fungi associated with *Dendroctonus* bark beetles can translocate nitrogen and phosphorus to increase their availability in the insect feeding sites (Six and Elser, 2020). However, direct fungus consumption by *I. typographus* requires further experimental confirmation.

In addition to fungi, bacteria isolated from conifer-feeding beetles and their galleries have been shown to regulate the growth and reproduction of the fungal partners (Adams et al., 2009), inhibit the growth of entomopathogenic fungi (Cardoza et al., 2006; Peral-Aranega et al., 2023), degrade terpenes (Adams et al., 2013; Boone et al., 2013; Xu et al., 2016; Berasategui et al., 2017), break down cellulose (Morales-Jimenez et al., 2012) and fix nitrogen (Morales-Jimenez et al., 2013). However, these potential benefits have not been tested experimentally *in beetlo*. Assessing the extent of these microbial contributions to host fitness requires the design of manipulative assays, which would allow testing the functional roles of the gut symbionts and their implications for their insect host (Ceja-Navarro et al., 2015; Dearing et al., 2022; Liu et al., 2022). Uncovering the functional importance of gut symbionts for *I. typographus* would provide us with a better understanding of this insect's ability to successfully invade chemically defended host trees. Improving our knowledge of this and other aspects of bark beetle ecology will help develop sustainable strategies to manage this pest in forests that are already challenged by a changing climate.

## Data availability statement

The datasets presented in this study can be found in online repositories. The names of the repository/repositories and accession number(s) can be found at: NCBI – accession PRJNA1060889, http://www.ncbi.nlm.nih.gov/bioproject/PRJNA1060889; Data repository of the Max Planck Society (“Edmond”): https://doi.org/10.17617/3.JXBYHO; GenBank accessions PP227293-PP227362: https://ncbi.nlm.nih.gov/nuccore.

## Author contributions

AB-Q: Conceptualization, Formal analysis, Investigation, Methodology, Visualization, Writing – original draft, Writing – review & editing. JG: Conceptualization, Funding acquisition, Methodology, Project administration, Supervision, Writing – review & editing. MK: Conceptualization, Funding acquisition, Methodology, Project administration, Supervision, Writing – review & editing.
