# Supplementary material for: The Eurasian spruce bark beetle Ips typographus shapes the microbial communities of its offspring and the gallery environment
Source: Front Microbiol. 2024 Feb 16;15:1367127. doi: 10.3389/fmicb.2024.1367127 (PMC10904642; doi:10.3389/fmicb.2024.1367127)
Supplement: Supplementary file 2 [file Table_2.PDF]

Supplementary table 2: statistical analysis of the beta diversity of the bacterial communities associated to the bark and the beetle-modified environment

PERMANOVA

	Df	SumOfSqs	R2	F	Pr(>F)
phyloseq::sample_data(ps_env_r)$stage	3	2.9242	0.32396	3.8337	0.001 ***
Residual	24	6.1021	0.67604		
Total	27	9.0262	1.00000		

Post-Hoc comparison using pairwiseAdonis

pairs	Df	SumsOfSqs	F.Model	R2	p.value	p.adjusted	
bark vs gallery	1	1.74184154	5.8753148	0.22706254	0.001	0.006	*
bark vs plugs	1	1.21152772	3.7084014	0.20941481	0.005	0.030	.
bark vs pupal_chamber	1	1.30003330	3.9861084	0.22162150	0.003	0.018	.
gallery vs plugs	1	0.10910809	0.7102883	0.06631832	0.602	1.000	
gallery vs pupal_chamber	1	0.14327572	0.9374780	0.08571245	0.432	1.000	
plugs vs pupal_chamber	1	0.05707452	1.3217203	0.24836336	0.300	1.000	

Supplementary table 3: statistical analysis of the beta diversity of fungal communities communities associated to the bark and the beetle-modified environment

PERMANOVA:

	Df	SumOfSqs	R2	F	Pr(>F)
phyloseq::sample_data(ps_env_r)$sampletype	3	2.8264	0.2935	3.3234	0.001 ***
Residual	24	6.8036	0.7065		
Total	27	9.6300	1.0000		

Post-Hoc comparison using pairwiseAdonis

pairs	Df	SumsOfSqs	F.Model	R2	p.value	p.adjusted	
bark vs gallery	1	1.3907	4.402	0.180	0.001	0.006	*
bark vs plugs	1	1.2754	4.016	0.222	0.003	0.018	.
bark vs pupal_chamber	1	0.9974	2.902	0.172	0.005	0.030	.
gallery vs plugs	1	0.4361	2.190	0.180	0.069	0.414	
gallery vs pupal_chamber	1	0.4450	1.890	0.159	0.114	0.684	
plugs vs pupal_chamber	1	0.266	2.194	0.354	0.100	0.600	

Significance codes: 0 ‘***’ 0.001 ‘**’ 0.01 ‘*’ 0.05 ‘.’ 0.1 ‘ ’ 1

Supplementary table 4: statistical analysis of the beta diversity of the bacterial communities associated to *Ips typographus* across life stages

PERMANOVA:

	Df	SumOfSqs	R2	F	Pr(>F)
phyloseq::sample_data(ps_transformb)$stage	4	2.535	0.136	2.636	0.001 ***
Residual	67	16.107	0.864		
Total	71	18.641	1.000		

Post-Hoc comparison using pairwiseAdonis

pairs	Df	SumsOfSqs	F.Model	R2	p.value	p.adjusted	
adult vs larva	1	0.8219395	3.148688	0.08958195	0.004	0.04	.
adult vs pupa	1	0.3051080	1.795383	0.06960093	0.071	0.71	
adult vs callow	1	0.6237147	2.593946	0.07721469	0.011	0.11	
adult vs egg	1	0.1659033	1.018133	0.07262971	0.414	1.00	
larva vs pupa	1	0.8659803	3.463823	0.09245780	0.006	0.06	
larva vs callow	1	0.7919739	2.734250	0.06251964	0.009	0.09	
larva vs egg	1	0.4321149	1.519669	0.06197756	0.148	1.00	
pupa vs callow	1	1.0293758	4.469156	0.11927560	0.001	0.01	*
pupa vs egg	1	0.2673157	1.770122	0.10555212	0.181	1.00	
callow vs egg	1	0.2894858	1.129077	0.04881633	0.324	1.00	

Significance codes: 0 ‘***’ 0.001 ‘**’ 0.01 ‘*’ 0.05 ‘.’ 0.1 ‘ ’ 1

Supplementary table 5: statistical analysis of the beta diversity of the fungal communities associated to *Ips typographus* across life stages

PERMANOVA:

	Df	SumOfSqs	R2	F	Pr(>F)
phyloseq::sample_data(ps_bb)$stage	4	3.5649	0.14769	2.9025	0.001 ***
Residual	67	20.5720	0.85231		
Total	71	24.1369	1.00000		

Post-Hoc comparison using pairwiseAdonis

pairs	Df	SumsOfSqs	F.Model	R2	p.value	p.adjusted	
pupa vs callow	1	1.6357647	4.956456	0.13058270	0.001	0.01	*
pupa vs adult	1	1.1461296	3.520219	0.12791393	0.002	0.02	.
pupa vs larva	1	1.6765561	5.260593	0.13399169	0.001	0.01	*
pupa vs egg	1	1.2796278	4.101048	0.21470278	0.003	0.03	.
callow vs adult	1	0.5423161	1.750219	0.05344144	0.061	0.61	
callow vs larva	1	0.3922343	1.273555	0.03012651	0.215	1.00	
callow vs egg	1	0.5604874	1.905278	0.07970115	0.071	0.71	
adult vs larva	1	0.4987348	1.671069	0.04962923	0.074	0.74	
adult vs egg	1	0.6262643	2.398055	0.15573755	0.029	0.29	
larva vs egg	1	0.5177328	1.855727	0.07465992	0.055	0.55	
