# Supplementary material for: The Eurasian spruce bark beetle Ips typographus shapes the microbial communities of its offspring and the gallery environment
Source: Front Microbiol. 2024 Feb 16;15:1367127. doi: 10.3389/fmicb.2024.1367127 (PMC10904642; doi:10.3389/fmicb.2024.1367127)
Supplement: Supplementary file 4 [file Data_Sheet_1.PDF]

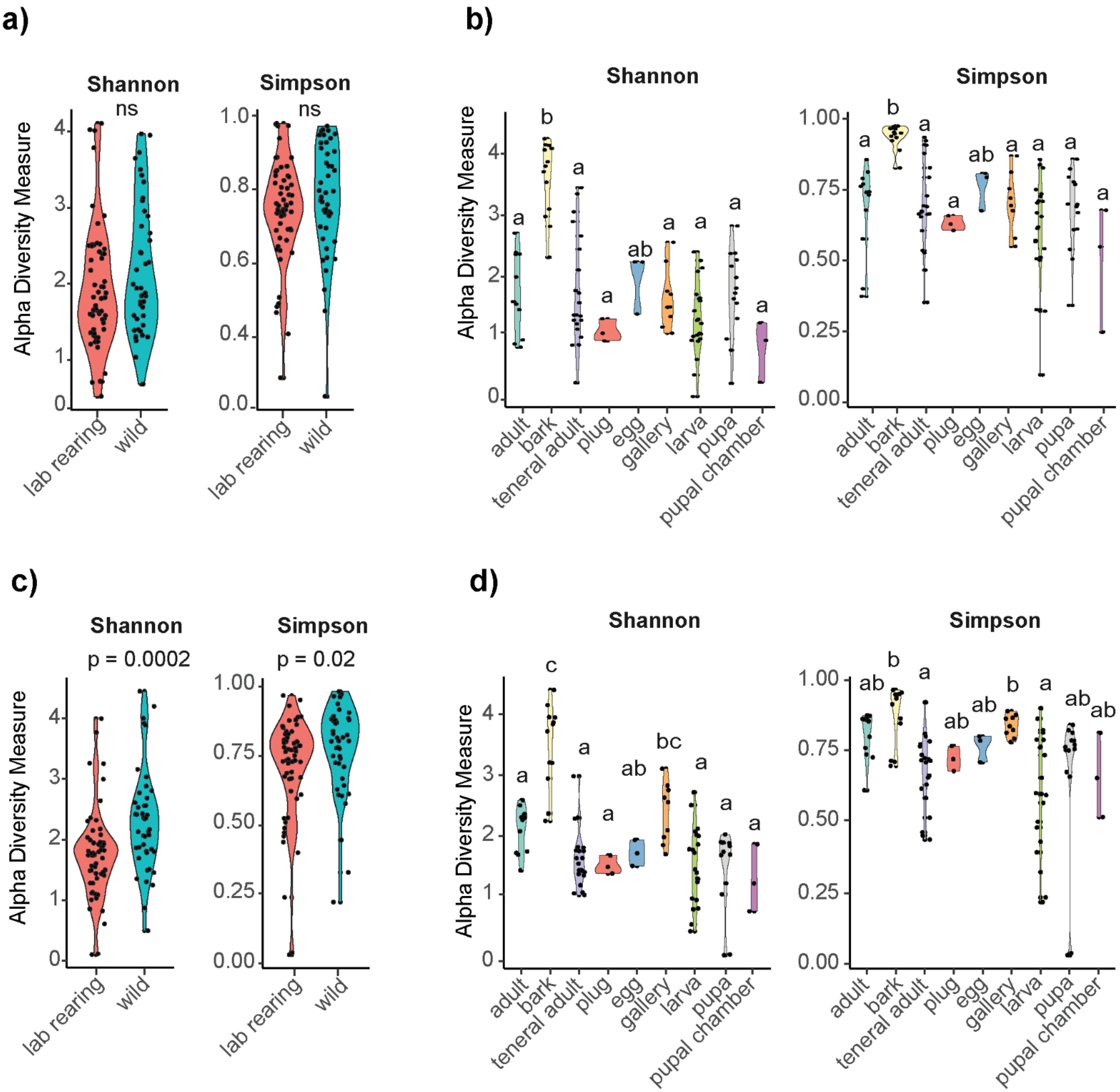

Supplementary figure 1. Bacterial and fungal alpha diversity. Shannon and Simpson indices of **a)** bacterial taxa in wild-caught vs. laboratory-reared beetles, **b)** bacterial taxa in different life stages, **c)** fungal taxa in wild-caught vs. laboratory-reared beetles, **d)** fungal taxa in different life stages. For a) and c) p-values of the Wilcoxon Rank Sum Test are shown, ns = non-significant ($p > 0.05$). For b) and d) different letters indicate significant differences among treatments (Kruskal-Wallis one-way analysis of variance, post-hoc Dunn's test with Bonferroni correction, $p < 0.05$).

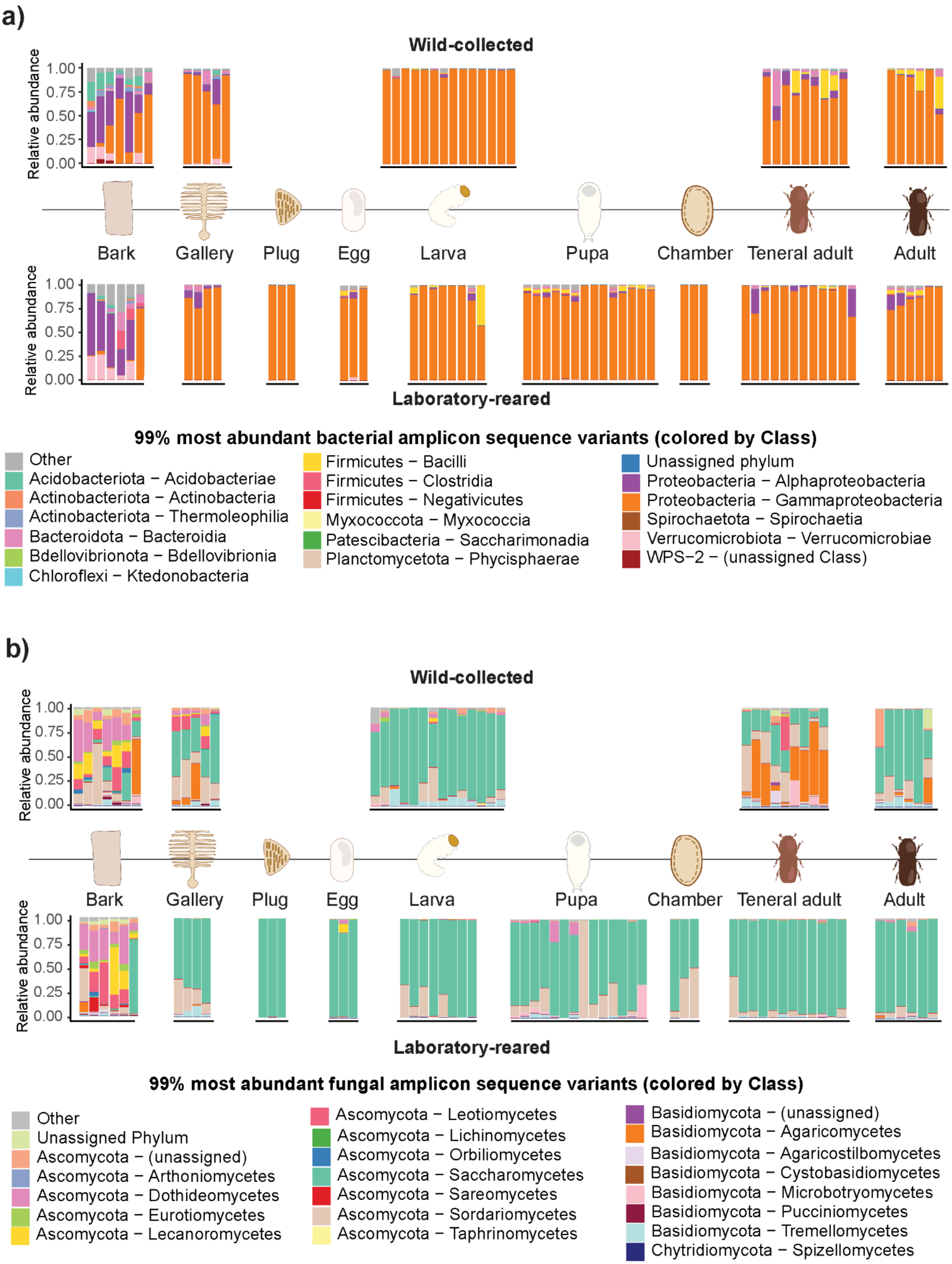

Supplementary figure 2. Relative abundances of the 99% most abundant amplicon sequence variants across all sample types. **a)** Dominant bacteria. **b)** Dominant fungi. Relative abundances indicated at Class level.

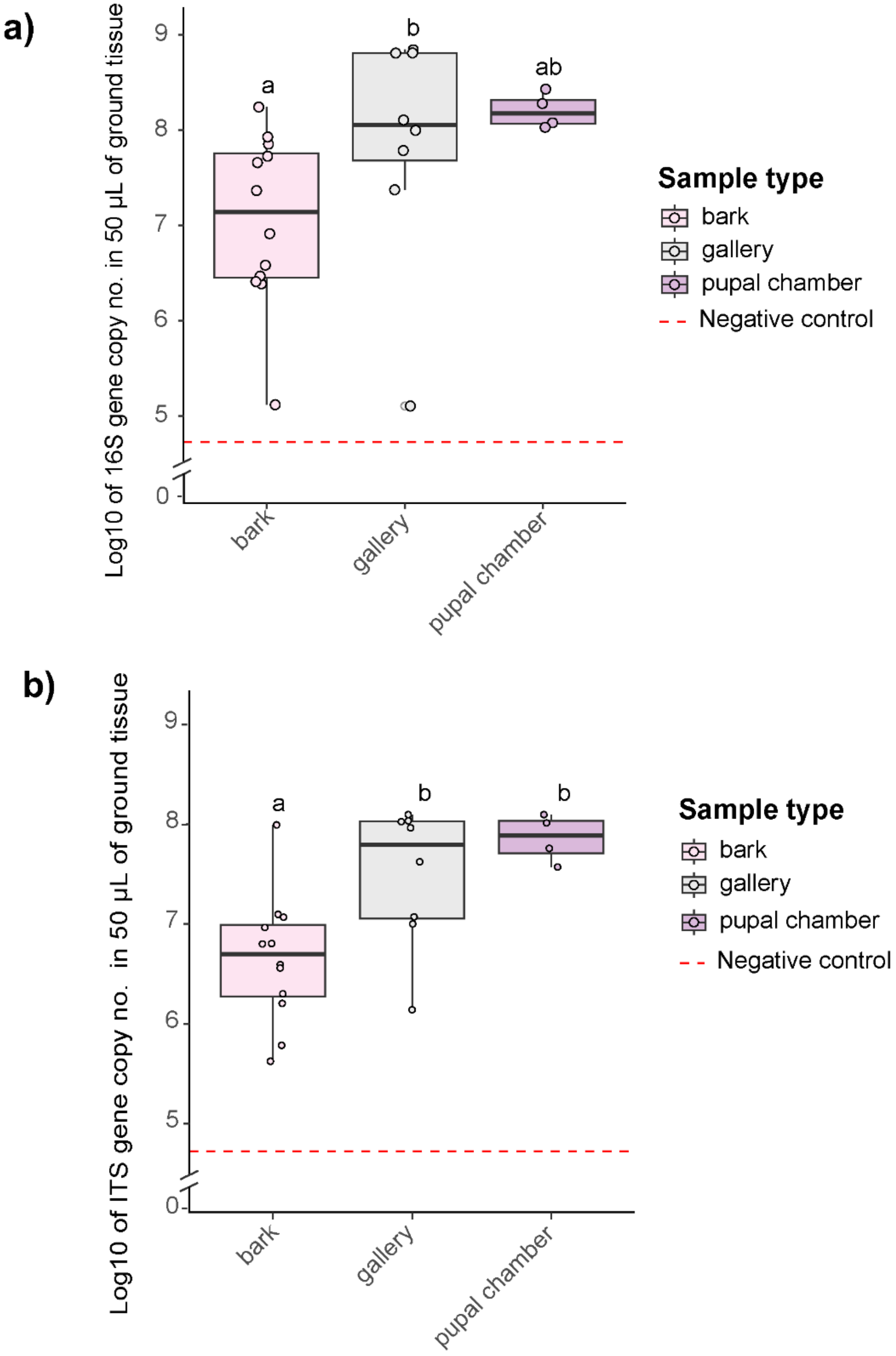

Supplementary figure 3. Microbial rRNA gene copy numbers in the gallery environment. **a)** Bacterial 16S gene copy numbers, **b)** Fungal ITS gene copy numbers. Negative controls were samples without any insect or plant tissue, but extraction reagents only, that were processed and subjected to quantitative PCR in the same manner as other samples. Letters indicate significant differences among groups (least-squares means pairwise comparison with Tukey adjustment, $p < 0.05$). Data were log-transformed to facilitate their visualization.

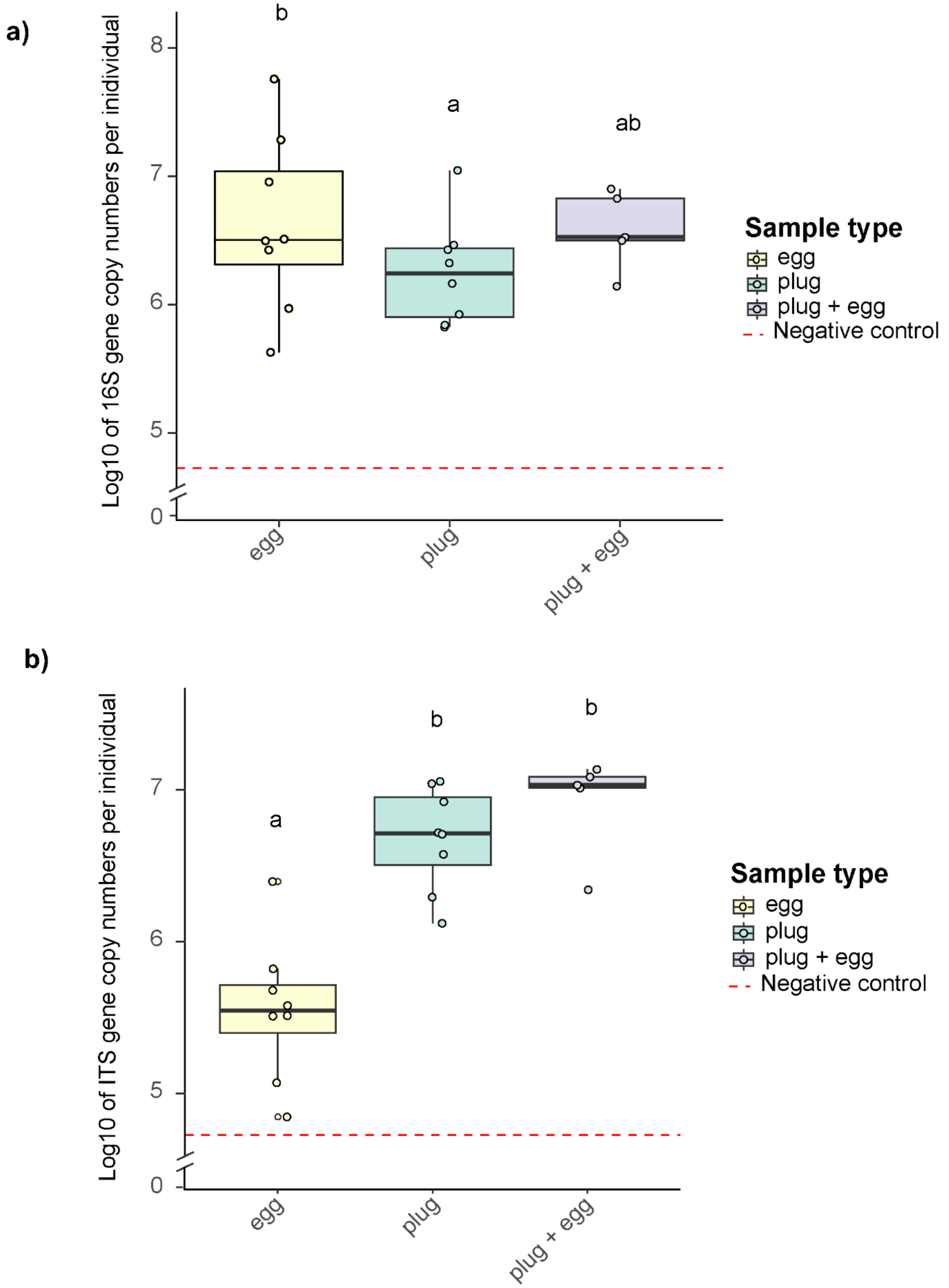

Supplementary figure 4. Microbial rRNA gene copy numbers in bark beetle eggs, egg plugs and eggs collected together with their plugs. **a)** Bacterial 16S gene copy numbers, **b)** Fungal ITS gene copy numbers. Negative controls were samples without any insect or plant tissue, but extraction reagents only, that were processed and subjected to quantitative PCR in the same manner as other samples. Letters indicate significant differences among groups (least-squares means pairwise comparison with Tukey adjustment, $p < 0.05$). Data were log-transformed to facilitate their visualization.

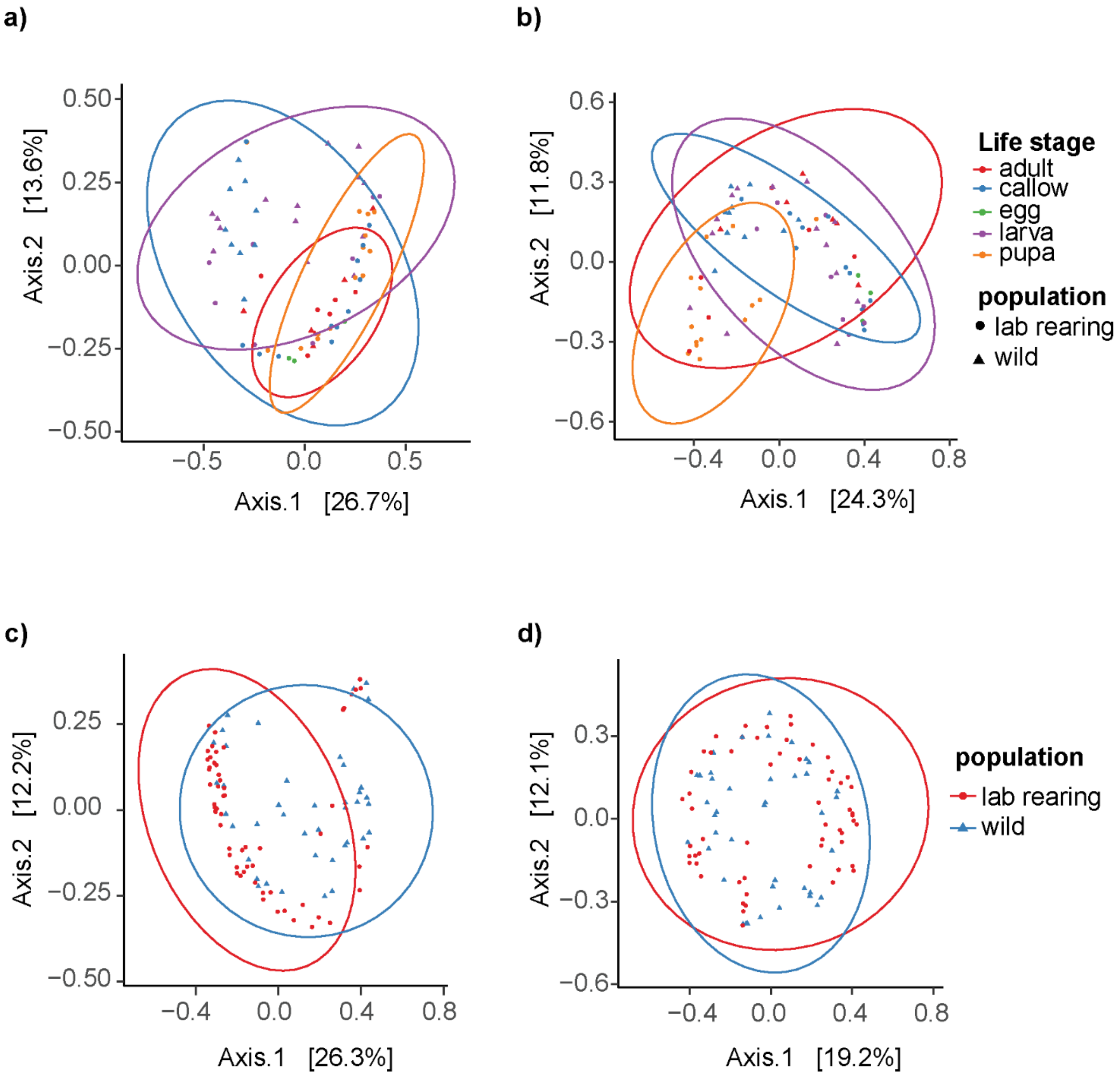

Supplementary figure 5. Beta diversity of bacterial and fungal communities associated with *I. typographus* and its environment in wild-collected and laboratory-reared samples. Principal Component Analysis (PCoA) plots of the Bray-Curtis distances for **a)** bacterial and **b)** fungal communities grouped by life stage **c)** bacterial and **d)** fungal communities grouped by population.
